# Analysis of *abrB* Expression during the Infectious Cycle of *Bacillus thuringiensis* Reveals Population Heterogeneity

**DOI:** 10.3389/fmicb.2017.02471

**Published:** 2017-12-12

**Authors:** Samia Ben Rejeb, Didier Lereclus, Leyla Slamti

**Affiliations:** Micalis Institute, Institut National de la Recherche Agronomique, AgroParisTech, Université Paris-Saclay, Jouy-en-Josas, France

**Keywords:** population heterogeneity, single cell analysis, *Bacillus thuringiensis*, infectious cycle, dormancy

## Abstract

Using the model host/pathogen pair *Galleria mellonella*/*Bacillus thuringiensis*, we have shown that these bacteria could kill their insect host, survive in its cadaver and form spores by sequentially activating virulence, necrotrophism and sporulation genes. However, the population isolated from the cadavers was heterogeneous, including non-sporulating cells in an unknown physiological state. To characterize these bacteria, we used a transcriptional fusion between the promoter of a gene expressed during early exponential growth (*abrB*) and a reporter gene encoding a destabilized version of GFP, in combination with a fluorescent reporter of the necrotrophic state. The composition of the bacterial population during infection was then analyzed by flow cytometry. We showed that the P*abrB* promoter was activated in the population that had turned on the necrotrophic reporter, suggesting a re-entry into vegetative growth. Strikingly, the cells that did not go through the necrotrophic state did not activate the P*abrB* promoter and appear as a dormant subpopulation. We propose a new model describing the *B. thuringiensis* cell types during infection.

## Introduction

Bacteria are often exposed to changes in their environment. Pathogenic bacteria in particular have to face an array of environments that change with the progression of the disease. They adapt most of the time by modifying their gene expression profile in response to the signals they receive. And interestingly, a clonal population will not always behave as a whole. Genetically identical bacteria can differentiate into specialized cell-types that will provide different answers to the signal received (Lopez and Kolter, 2010; Verplaetse et al., 2015; Mouammine et al., 2017).

Our model bacteria belong to the *Bacillus cereus* group. This group is composed of 8 Gram-positive and sporulating species including *B. anthracis*, *B. thuringiensis* and *B. cereus sensu stricto*. These pathogens are responsible for infections in humans and in animals. *B. anthracis* is the agent of anthrax (Mock and Fouet, 2001). *B. cereus* mainly causes food-borne toxi-infections and is also an opportunistic pathogen responsible of endocarditis, meningitis and endophthalmitis (Stenfors Arnesen et al., 2008; Bottone, 2010). *B. thuringiensis* is an insect pathogen whose host specificity depends on the production of insecticidal toxins (Deng et al., 2014). The entomopathogenic properties of this bacterium are widely used in the world for pest control (Sanahuja et al., 2011; Sanchis, 2011).

To understand the mechanisms involved in the pathogenicity of these bacteria we work with the insect model *Galleria mellonella*. This infection model has been successfully used to characterize numerous genes involved in the pathogenic properties of *B. cereus* and *B. thuringiensis* (Salamitou et al., 2000; Fedhila et al., 2006; Raymond et al., 2010). These bacteria are capable of carrying out a full infectious cycle in the larva of this insect. We have shown that this process is composed of three major phases (for a review Slamti et al., 2014). At the beginning of the infection, virulence factors are expressed under the control of the quorum sensor PlcR. These factors, which include proteases, phospholipases and cytotoxins, allow the bacteria to invade its host and to kill it (Salamitou et al., 2000). After the death of the insect, NprR, another quorum sensor, will trigger a necrotrophic lifestyle permitting the bacteria to survive in the cadaver (Dubois et al., 2016). Finally, the cells will sporulate and will be able to disseminate and withstand hostile environmental conditions.

A recent study reported the differentiation process of cells in insect cadavers, using fluorescent reporters under the control of promoters reflecting the activity of the regulators responsible for virulence, necrotrophism and sporulation at the cell level (Verplaetse et al., 2015). Although these physiological states take place in a sequential manner in a cell, the necrotrophic phase is only triggered in a part of the population in the insect cadaver, suggesting an activation specificity linked to the environment. It was also shown that sporulation only occurs in the sub-population that has activated the necrotrophic regulon. This is in agreement with the fact that the apo form of NprR inhibits sporulation, whereas NprR in complex with its signaling peptide NprX, activates the necrotrophic genes (Perchat et al., 2016). We also identified a category of cells that did not express any of the previously described reporters in biofilm and in the host (Verplaetse et al., 2015, 2016). This category represented about 20% of the population throughout the infectious process.

Here we investigated the physiological state of the cells that did not enter the necrotrophic state, since the necrotrophic sub-population is the most inclusive. We sought to determine if these cells were actively growing bacteria. To monitor the vegetative state of these bacteria we chose to assay the activity of the promoter of the *abrB* gene during insect infection using a fluorescent reporter. This gene encodes the AbrB transition state regulator and is transcribed during the transition from lag to exponential phase and during early exponential phase (O’Reilly and Devine, 1997; Lucking et al., 2009). This central regulator has been shown to repress the expression of stationary phase genes in *B. subtilis* (Perego et al., 1988; Strauch and Hoch, 1993) as well as the synthesis of the cereulide toxin synthesis and the expression of the *inhA1* metalloprotease-encoding gene in *B. cereus* (Grandvalet et al., 2001; Lucking et al., 2009). We engineered a *B. thuringiensis*-improved allele of the bright and fast folding sfGFP (Pedelacq et al., 2006) and, in order to follow fluctuations in gene expression, we destabilized the resulting protein by using the *ssrA*-mediated peptide tagging system that addresses specifically tagged proteins to the Clp degradation machinery in *Escherichia coli* and in *B. subtilis* (Keiler et al., 1996; Gottesman et al., 1998; Wiegert and Schumann, 2001). Using this tool, we showed that the *abrB* gene is expressed at the beginning of the infection and at a later stage of the process, only in cells that already went through the necrotrophic state. However, there is still a bacterial population in an unknown physiological state and we show that more than 60% of this population are living cells.

## Materials and Methods

### Bacterial Strains and Growth Conditions

The acrystalliferous *B. thuringiensis* 407 Cry^-^ strain (Bt 407^-^) (Lereclus et al., 1989) was used as the parental strain to create all the strains used in this study. *E. coli* strain DH5α (Taylor et al., 1993) was used as the host strain for plasmid construction. *E. coli* strain ET12567 (MacNeil et al., 1992) was used to prepare DNA prior to electroporation in *B. thuringiensis*. Cells were grown in LB medium (1% tryptone, 0.5% yeast extract, 1% NaCl) or HCT medium (0.7% casein hydrolysate, 0.5% tryptone, 0.68% KH2PO4, 0.012% MgSO4 7H2O, 0.00022% MnSO4 4H2O, 0.0014% ZnSO4 7H2O, 0.008% ferric ammonium citrate, 0.018% CaCl2 4H2O, 0.3% glucose, pH 7.2) (Lereclus et al., 1982) at 37°C and stored at -80°C in LB containing 15% glycerol.

For *B. thuringiensis* cultures, t0 corresponds to the beginning of the transition between the exponential and stationary phases of growth.

The antibiotic concentrations used for selection of *B. thuringiensis* and *E. coli* were as follows: erythromycin, 10 μg/mL; ampicillin, 100 μg/mL. Chloramphenicol was used at a concentration of 100 μg/mL to block the synthesis of proteins (Periago et al., 2002) in *B. thuringiensis*.

When required, xylose was used at a concentration of 20 mM.

### Plasmid and Strain Constructions

DNA manipulations are detailed in the Supplementary Experimental Procedures. All the plasmids and strains used in this study are indicated in **Tables 1**, **2**. Oligonucleotides are listed in Supplementary Table S1.

**Table 1:** Plasmids used in this study.

Name	Relevant information	Reference
pHT304	Replicative multicopy *E. coli*/*B. thuringiensis* shuttle vector.	Arantes and Lereclus, 1991
pHT304.18	Replicative multicopy *E. coli*/*B. thuringiensis* shuttle vector.	Agaisse and Lereclus, 1994
p304-P*xyl*+	pHT304 harboring a modified version of the xylose-inducible promoter region of *xylA* to enhance translation efficiency (Stammen et al., 2010). The original sequence **AGGGGG**AATCACATG was changed to **AGGAGG**TGACACCATG were the RBS is in bold letters and the translation start site is underlined.	Slamti et al., 2015
pPx’*_sf_gfp*	*_sf_gfp* was amplified by PCR from pCM11 (Pedelacq et al., 2006) using primer pair sfgfp1/sfgfp2, digested with BglII and KnpI, and cloned between the BamHI and KpnI restriction sites of pHT304.18-Px (Slamti and Lereclus, 2002). This created a transcriptional fusion between the xylose-inducible promoter region of *xylA* and *_sf_gfp*.	This study
pPx’*gfp_Bt_*	*B. thuringiensis* codon optimized *gfp_Bt_*, synthesized and cloned in the pEX plasmid by Eurofins Genomics (France), was amplified by PCR from this vector using primer pair sfgfp1/sfgfpBt2, digested with BglII and KnpI, and cloned between the BamHI and KpnI restriction sites of pPx to create a transcriptional fusion between P*xylA* and *gfp_Bt_*.	This study
pPx+’*gfp_Bt_*	*B. thuringiensis* codon optimized *gfp_Bt_*, synthesized and cloned in the pEX plasmid by Eurofins Genomics (France), was amplified by PCR from this vector using primer pair sfgfpBt1/sfgfpBt2, digested with BsaI and KpnI, and cloned between the NcoI and KpnI restriction sites of p304-P*xyl*+ to create a transcriptional fusion between P*xyl*+ and *gfp_Bt_*.	This study
pPx’*gfp_Bte_*	*B. thuringiensis* codon optimized *gfp_Bt_*, synthesized and cloned in the pEX plasmid by Eurofins Genomics (France), was amplified by PCR from this vector using primer pair sfgfpBtcomGBt1/sfgfpBt2, digested with BsaI and KpnI, and cloned between the NcoI and KpnI restriction sites of p304-P*xyl*+. The forward primer included 24 bp encoding the first eight amino acids of *comGA*. These have been shown to enhance the stability of fluorescent proteins (Veening et al., 2004). The resulting cassette comprising the modified RBS from P*xyl*+ and the *comGA*’*gfp_Bt_* sequence was designated *gfp_Bte_*.	This study
pPx’*gfp_Bte_*LAA/ LVA/AAV/ASV	*gfp_Bte_* was amplified by PCR from pPx’*gfp_Bte_* using primer pairs sfgfpBtcomGBt1/gfpLAA-gfpLVA-gfpAAV-gfpASV, digested with BsaI and KpnI, and cloned between the NcoI and KpnI restriction sites of p304-P*xyl*+. The reverse primers add an *ssrA* tag to the gene: GKQNNLLSLAA for *gfp_Bte_*LAA, GKQNNLLSLVA for *gfp_Bte_*LVA, GKQNNLLSAAV for *gfp_Bte_*AAV and GKQNNLLSASV for *gfp_Bte_*ASV. These tags will address the protein to proteases that will degrade them with varying efficiencies (Keiler and Sauer, 1996; Keiler et al., 1996; Andersen et al., 1998).	This study
pHT-*gfp_Bte_*AAV	*gfp_Bte_*AAV was amplified by PCR from pPx’*gfp_Bte_*AAV using primer pair Xyl10/PU, and cloned between the SmaI and EcoRI restriction sites of pHT304.18.	This study
pP*abrB*’*gfp_Bte_*AAV	The promoter region of the *abrB* gene was amplified by PCR from the chromosome of Bt 407 using primer pairs PabrB-F-XbaI/PabrB-R-AscI and cloned between the XbaI and AscI restriction sites of pHT-*gfp_Bte_*AAV.	This study
pP*nprA*’*mcherry_LGC_*	pHT304.18 harboring a transcriptional fusion between the promoter of *nprA* and the *B. thuringiensis*-adapted *mcherry* reporter gene.	Verplaetse et al., 2015
pP*abrB*’*gfp_Bte_*AAV-P*nprA*’*mcherry_LGC_*	The transcriptional fusion between the promoter region of *abrB* and the promoterless *gfp_Bte_*AAV reporter gene was amplified from pP*abrB*’*gfp_Bte_*AAV using primer pair PabrB-F-NcoI/gfpBteAAVin-BglII. pP*spoIID*’*yfp*-P*nprA*’*mcherry_LGC_* (Verplaetse et al., 2015) was used as a PCR template to amplify the plasmid without the P*spoIID*’*yfp* region using primer pair Term-R-BglII/NprA-F-NcoI. This fragment and P*abrB*’*gfp_Bte_*AAV were digested with BglII and NcoI and ligated together to generate pP*abrB*’*gfp_Bte_*AAV-P*nprA*’*mcherry_LGC_* which harbors the two transcriptional fusions in divergent orientation and separated by about 100 bp.	This study
pP*aphA3*’*_sf_gfp*	*_sf_gfp* was amplified by PCR from pCM11 (Pedelacq et al., 2006) using primer pair sfgfp1/sfgfp2 and digested with BglII and EcoRI. P*aphA3* was amplified from pDG792 (Guerout-Fleury et al., 1995) using primer pair PkanHind1/PkanBam2 and digested with HindIII and BamHI. Both fragments were cloned between the HindIII and EcoRI restriction sites of pHT304.18 generating a transcriptional fusion between the constitutive P*aphA3* promoter and *_sf_gfp*.	This study

**Table 2:** Strains used in this study.

Name	Relevant information	Reference
Bt (pHT304)	Bt 407^-^ carrying the empty pHT304 vector and used as a Fluorescence^-^ control.	This study
Bt (pPx)	Bt 407^-^ carrying the empty pPx vector and used as a Fluorescence^-^ control.	This study
Bt (pPx’*_sf_gfp*)	Bt 407^-^ used to measure the fluorescence generated by the transcriptional fusion between the xylose-inducible promoter of *xylA* and *_sf_gfp*.	This study
Bt (pPx’*gfp_Bt_*)	Bt 407^-^ used to measure the fluorescence generated by the transcriptional fusion between the xylose-inducible promoter of *xylA* and the *B. thuringiensis* codon-optimized *gfp_Bt_*.	This study
Bt (pPx+’*gfp_Bt_*)	Bt 407^-^ used to measure the fluorescence generated by the transcriptional fusion between P*xyl*+ and the *B. thuringiensis* codon-optimized *gfp_Bt_*.	This study
Bt (pPx’*gfp_Bte_*)	Bt 407^-^ used to measure the fluorescence generated by the transcriptional fusion between P*xyl*+ and the *B. thuringiensis* codon-optimized *gfp_Bt_* to which the sequence encoding the first eight amino acids of *comGA* have been added.	This study
Bt (pPx’*gfp_Bte_*LAA/ LVA/AAV/ASV)	Bt 407^-^ used to measure the fluorescence generated by the transcriptional fusion between P*xyl*+ and *gfp_Bte_* to which a degradation tag has been added.	This study
Bt (pP*abrB*’*gfp_Bte_*AAV)	Bt 407^-^ in which we measure the activity of the promoter of *abrB* using a reporter gene encoding an unstable GFP.	This study
Bt (pP*nprA*’*mcherry_LGC_*)	Bt 407^-^ in which we measure the activity of the promoter of *nprA* using the *mcherry* reporter gene.	Verplaetse et al., 2015
Bt (pP*abrB*’*gfp_Bte_*AAV-P*nprA*’*mcherry_LGC_*)	Bt 407^-^ in which we measure the activity of the promoter of *abrB,* using a reporter gene encoding an unstable GFP, as well as the activity of the promoter of *nprA*, using *mcherry*.	This study
Bt (pP*aphA3*’*_sf_gfp*)	Bt 407^-^ used to measure the fluorescence generated by the transcriptional fusion between the promoter of *aphA3* and *_sf_gfp*.	This study

### *In Vitro* Growth of the Cells for Measurement of the GFP-Based Fluorescence

To assay the GFP-based fluorescence of cells harboring the *_sf_gfp* gene and its derivatives designed to improve fluorescence, overnight cultures incubated at 30°C in HCT medium supplemented with erythromycin were diluted 1000-fold in HCT and incubated at 37°C under agitation until an OD_600_ of 0.5. Xylose was then added and growth was resumed. Cells were harvested at the time of xylose addition (T0) as well as at other time points after xylose addition (Tn). Cultures were carried out in the same way to assay the GFP-based fluorescence of cells harboring the *gfp_Bte_* gene and its derivatives designed to destabilize the GFP, except that chloramphenicol was added to the cells 1 h after xylose addition.

In all cases, the cells were harvested and fixed as described in Verplaetse et al. (2015). Essentially, the cells were centrifuged, fixed for 7 min in PBS-formaldehyde 4% then washed in PBS. The pellet was then resuspended in GTE buffer (Vlamakis et al., 2008) and kept at 4°C until flow cytometry analysis or microscopy.

### *In Vivo* Experiments

Intrahemocoelic injection experiments with *G. mellonella* were carried out essentially as described previously (Salamitou et al., 2000; Verplaetse et al., 2015). For each strain, 20 larvae were injected each with 2.10^4^ bacteria and kept at 30°C for 72 h. 18 h after injection, surviving insects were eliminated. At each time point, *B. thuringiensis* cells were harvested from 2 or 3 dead insects as follows: the larva was cut open, transferred to a 1.5 mL Eppendorf tube containing 1 ml of PBS and vortexed. The suspension was pipetted into a new 1.5 mL Eppendorf tube (leaving behind most of the large insect debris). The sample was centrifuged and the pellet was resuspended in PBS-formaldehyde 4%, fixed for 7 min then washed in PBS. This suspension was filtered onto a cotton pad in a 1 mL syringe in order to retain the cadaver debris and recover the bacterial cells in the filtrate. These were then concentrated by centrifugation, resuspended in GTE buffer (Vlamakis et al., 2008) and kept at 4°C until flow cytometry analysis or microscopy.

For the assessment of the percentage of live cells in the bacterial population in insect cadavers, the experiments were carried out in the same manner except that the cells were not fixed with formaldehyde. After filtration on the cotton pad, the suspension was centrifuged, resuspended in saline, incubated with the SYTOX Green Dead Cell Stain (Molecular Probes) according to the manufacturer’s instructions and analyzed immediately with the flow cytometer. Dead bacteria will present a bright green fluorescence compared to live cells.

### Flow Cytometry Analysis

Fluorescence was measured on a CyFlow Space cytometer (Partec, France). Details about the parameters used to collect fluorescence and the softwares used to analyze the data are given in the Supplementary Experimental Procedures. The different populations were identified using histograms or bi-parametric cytograms. GFP- or mCherry-expressing cells were identified as cells giving a higher signal intensity than the reporterless cells used as controls.

### Fluorescence Microscopy

Cells were observed with an AxioObserver.Z1 Zeiss inverted fluorescence microscope equipped with a Zeiss AxioCam MRm digital camera and with Zeiss fluorescence filters. GFP was imaged using the 38 HE filter (excitation: BP 470/40, beam splitter: FT 495, emission: 525/50). mCherry was imaged using the 45 HE filter (excitation: BP 590/20, beam splitter: FT 605, emission: 620/14). Images were processed using the ZEN software package.

### Nucleotide Sequence Accession Numbers

The DNA sequence of the plasmid containing the *gfp_Bte_*AAV sequence was submitted to GenBank and is available under the accession number MF673728.

## Results

### Construction of a GFP Variant Highly Expressed in *B. thuringiensis*

**FIGURE 1:**
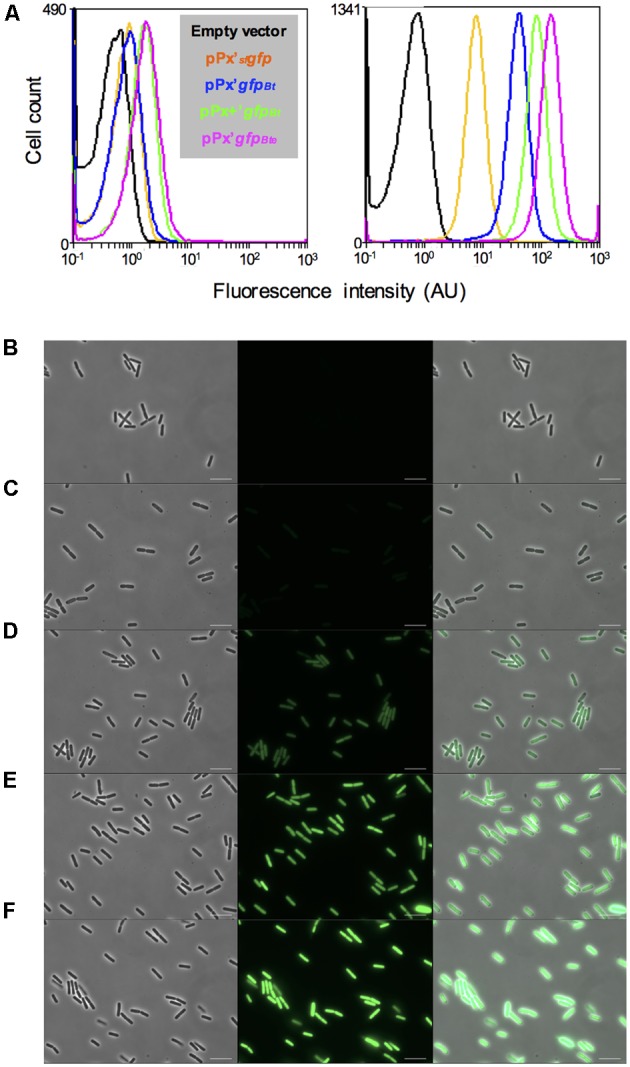
Comparison of the GFP-based fluorescence of *Bacillus thuringiensis* strains. **(A)** Flow cytometry analysis of the *B. thuringiensis* cells harboring the empty vector p304-P*xyl*+ (black), pPx’*_sf_gfp* (orange), pPx’*gfp_Bt_* (blue), pPx+’*gfp_Bt_* (green) or pPx’*gfp_Bte_* (pink). The left and right panels represent the fluorescence of the cells harvested when xylose was added and 2 h after xylose addition, respectively. The *X*-axis of the histograms is the fluorescence intensity in arbitrary units (AU) in logarithmic scale. The *Y*-axis represents the cell count. **(B–F)** Fluorescence microscopy images of cells harboring p304-P*xyl*+ **(B)**, pPx’*_sf_gfp* **(C)**, pPx’*gfp_Bt_* **(D)**, pPx+’*gfp_Bt_* **(E)**, or pPx’*gfp_Bte_* **(F)**. The cells were harvested 2 h after xylose addition. Left panels, phase contrast images; middle panels, epifluorescence images; right panels, merge between the two channels. The scale bars represent 10 μm. These results are representative of two independent experiments.

To facilitate the analysis of gene expression in *B. thuringiensis*, we engineered a highly expressed version of the sfGFP. We used the reporterless *_sf_gfp* (Pedelacq et al., 2006) under the control of the xylose-inducible P*xylA* promoter as a template for our improvement procedure. *_sf_gfp* encodes a version of the *Aequorea victoria* GFP that has been shown to be four times brighter and that folds 4 times faster *in vivo* than GFPmut3 (Milde, 2008). We modified the *_sf_gfp* gene and the features required for its translation to adapt it to *B. thuringiensis*. Our first step was to change the codon usage to one that would correspond better to that of our strain, using the GENEius software and the associated *B. thuringiensis* serovar *thuringiensis* codon usage table (Eurofins Genomics). We designated this allele *gfp_Bt_*. We then modified the RBS present in the promoter region of P*xylA* to the improved RBS+, that resulted in a twofold increase in protein production in *B. megaterium*, as described in Stammen et al. (2010). Finally, we added the first 24 bp of *comGA* to the 5’ end of the *_sf_gfp* coding sequence. The corresponding eight amino acids have been shown to enhance the stability of fluorescent proteins (Veening et al., 2004). The resulting cassette comprising the modified RBS and the *comGA*’*gfp_Bt_* sequence was designated *gfp_Bte_*. Using flow cytometry, we showed that the *gfp_Bte_*-expressing cells grown in HCT medium were about 20 times brighter than the *_sf_gfp*-expressing cells, 2 h after the addition of xylose (**Figure 1A**; median fluorescence of 34 and 1.6 AU and pink and orange lines, respectively). Codon adaptation resulted in the most efficient improvement compared to the RBS modification and the *comGA* sequence addition. Indeed, the *gfp_Bt_*-expressing cells are fivefold brighter than the *_sf_gfp*-expressing cells, whereas each subsequent modification step increased the fluorescence of the cells by approximately twofold (**Figure 1A**; median fluorescence of 8, 1.6, 22, and 34 AU and blue, orange, green and pink lines, respectively). This increase in fluorescence intensity is visible on the microscopy pictures taken 2 h after xylose addition (**Figures 1B–F**).

### Construction of Unstable GFP_Bte_ Variants

**FIGURE 2:**
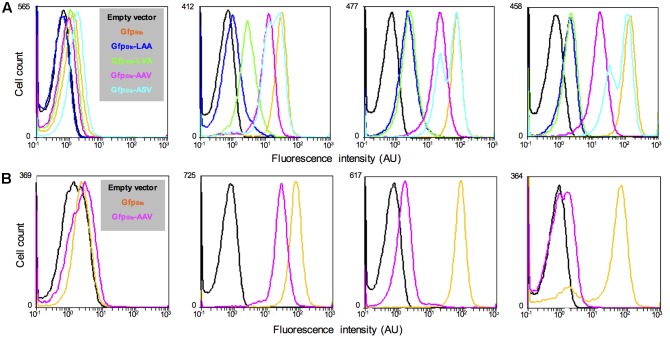
Comparison of the fluorescence of *B. thuringiensis* strains harboring different alleles of *gfp_Bte_*. **(A)** Flow cytometry analysis of the *B. thuringiensis* cells harboring the empty vector p304-P*xyl*+ (black), pPx’*gfp_Bte_* (orange), pPx’*gfp_Bte_*LAA (blue), pPx’*gfp_Bte_*LVA (green), pPx’*gfp_Bte_*AAV (pink), or pPx’*gfp_Bte_*ASV (turquoise). The cells were harvested when xylose was added, 30 min, 1 h and 2 h after xylose addition (panels from left to right). **(B)** Flow cytometry analysis of the *B. thuringiensis* cells harboring the empty vector p304-P*xyl*+ (black), pPx’*gfp_Bte_* (orange), and pPx’*gfp_Bte_*AAV (pink). From left to right, the histograms show the fluorescence of cells harvested when xylose was added, 1 h after xylose addition (which also corresponds to the time when chloramphenicol was added), 1 and 22 h after chloramphenicol addition. The *X*-axis of the histograms is the fluorescence intensity in arbitrary units (AU) in logarithmic scale. The *Y*-axis represents the cell count. These results are representative of two independent experiments.

To monitor variations in gene expression, we decided to generate an unstable version of the GFP_Bte_. We used a previously described method that takes advantage of the *ssrA*-mediated peptide tagging system (Andersen et al., 1998). The principle of the method is to tag a protein for specific degradation by a protease. We added 10 amino acids at the N-terminal end of GFP_Bte_ with a variation in the last 3 amino acids of the sequence (GKQNNLSLAA/-LVA/-AAV/-ASV), generating GFP_Bte_LAA, GFP_Bte_LVA, GFP_Bte_AAV, and GFP_Bte_ASV. Amino acids GKQNNLSLAA correspond to the putative proteolytic tag added by the *ssrA* tmRNA we identified in the genome of strain *B. thuringiensis* 407. These amino acids have been shown to address the tagged protein to the Clp degradation machinery in *E. coli* and in *B. subtilis* (Keiler et al., 1996; Gottesman et al., 1998; Wiegert and Schumann, 2001). Each tag variant should alter the stability of the protein (Keiler and Sauer, 1996; Andersen et al., 1998). We monitored the fluorescence of the cells harboring the GFP_Bte_ tagged variants during growth in HCT medium and the results are presented on **Figure 2**. At the time of inducer addition, all the cells present a fluorescence similar to that of the reporterless cells (**Figure 2A**, left panel). 30 min after xylose induction, there is a striking difference between the cells harboring the wild-type -LAA or the -LVA tag and the cells harboring the -AAV or the -ASV tag (**Figure 2A**, second panel from the left). The latter present a fluorescence closer to that of the cells producing GFP_Bte_ whereas the fluorescence of the former is weaker. 1 h after xylose induction (**Figure 2A**, third panel from the left), the cells harboring the GFP_Bte_LAA or the GFP_Bte_LVA tag had a similar fluorescence (median fluorescence of 2 and 2.3 AU which is 33- and 28-fold lower than that of GfpBte-producing cells, respectively). The cells expressing *gfp_Bte_*ASV were the most fluorescent (median fluorescence of 43AU, 1.5-fold lower than that of *gfp_Bte_*-expressing cells), however, the histogram profile was not satisfying since it showed two peaks which indicate that *gfp* expression and/or GFP degradation in the population was highly heterogeneous. The cells harboring the GFP_Bte_AAV presented a median fluorescence of 19 AU that was 3.5-fold lower than that of the GFP_Bte_-producing cells (median fluorescence of 65 AU) and a histogram profile similar to that of the latter. Two hours after addition of the inducer (**Figure 2A**, right panel), all the histograms were similar to those observed 1 h after induction. *gfp_Bte_*-expressing cells were more fluorescent than 1 h before (median fluorescence of 125 AU vs. 65 AU, respectively) whereas the fluorescence of *gfp_Bte_*AAV-expressing cells was similar at both times (median fluorescence of 19 AU and 15 AU 1 and 2 h after addition of xylose, respectively). *gfp_Bte_*AAV was thus chosen as a reporter of gene expression for the subsequent experiments.

The decrease in fluorescence of GFP_Bte_AAV-producing cells was then monitored after the addition of chloramphenicol, an inhibitor of translation (Periago et al., 2002), to determine GFP stability (**Figure 2B**). The median fluorescence of *gfp_Bte_*-expressing cells was similar at the time of and 1 h after chloramphenicol addition (median fluorescence of 73 AU and 77 AU, respectively) (**Figure 2B**, second and third panels from the left, respectively). In contrast, the median fluorescence of *gfp_Bte_*AAV-expressing cells decreased by 17-fold between these two time-points (median fluorescence of 26 AU and 1.5 AU, respectively). 22 h after chloramphenicol addition, the majority of the *gfp_Bte_*-expressing cells remained fluorescent whereas the *gfp_Bte_*AAV-expressing cells presented a fluorescence close to that of the control cells (**Figure 2B**, right panel). These results indicate that, in these conditions, the GFP_Bte_AAV-based fluorescence shows a half-life of approximately 15 min.

### Monitoring the Expression of *abrB* *in Vitro* Using *gfp_Bte_*AAV

**FIGURE 3:**
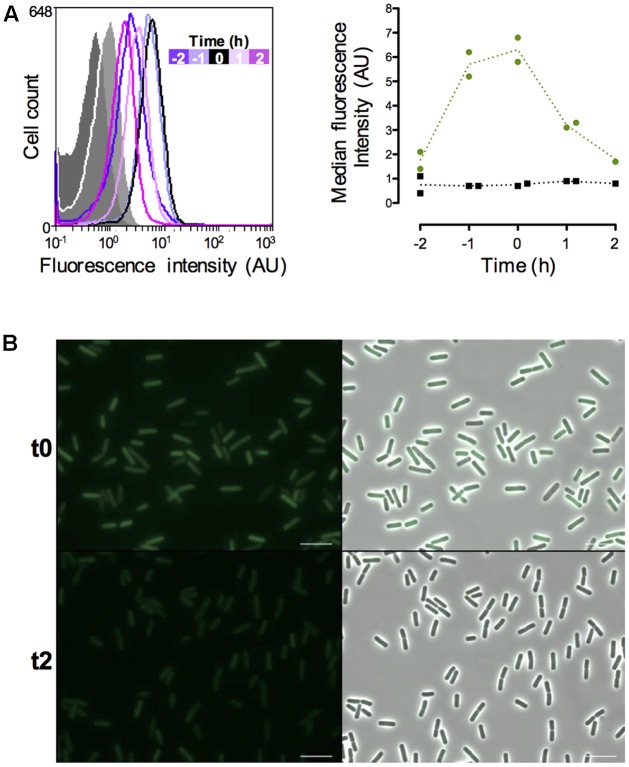
Expression of the *abrB*-driven *gfp_Bte_*AAV gene *in vitro*. **(A)** Left panel: flow cytometry analysis of the *B. thuringiensis* cells harboring the empty vector pHT304 (gray areas) or pP*abrB*’*gfp_Bte_*AAV (lines). The cells were grown in LB and harvested at various times during growth that can be identified with the time map on the graph. The negative control cells represented here were harvested at t-2 (dark gray) and t2 (light gray). The *X*-axis of the histograms is the fluorescence intensity in arbitrary units (A.U.) in logarithmic scale. The *Y*-axis represents the cell count. These results are representative of two independent experiments. Right panel: median fluorescence intensity of the same cells represented as a function of time. Black squares, *B. thuringiensis* cells harboring the empty vector pHT304; Green dots, *B. thuringiensis* cells harboring pP*abrB*’*gfp_Bte_*AAV. Each dot/square represents a replicate. The dotted lines connect the mean values at each time point. **(B)** Fluorescence microscopy images of cells harboring pP*abrB*’*gfp_Bte_*AAV at t0 and t2. Left panels, epifluorescence images; right panels, merge between the two channels. The scale bar represents 10 μm. t0 corresponds to the beginning of the transition between the exponential and stationary phases. These results are representative of two independent experiments.

In order to monitor the vegetative state of *B. thurin*giensis cells during the infectious cycle we chose to use the promoter of the *abrB* gene. This gene encodes a transition state regulator transcribed and active during the exponential phase (Perego et al., 1988; O’Reilly and Devine, 1997; Banse et al., 2008; Lucking et al., 2009). We monitored the fluorescence of the cells harboring a P*abrB*’*gfp_Bte_*AAV transcriptional fusion during growth in LB medium and the results are presented on **Figure 3**. The data are from two independent experiments. We represented 2 negative control samples on the graph to account for the difference between the auto-fluorescence of the reporter-less cells at t-2 and the cells harvested at the other time-points (**Figure 3A**, left panel). All the negative control histograms are shown in Supplementary Figure S1. The flow cytometry histograms show that the P*abrB*’*gfp_Bte_*AAV-expressing cells at t-2 are already fluorescent compared to the negative control (median fluorescence of 2.1 vs. 0.4 AU, respectively) and their fluorescence increases until it reaches a maximum at t-1 and t0 before decreasing at t1 and t2 (median fluorescence of 5.2, 5.8, 3.1, and 2.7 AU, respectively, to be compared to the light gray negative control with a median fluorescence of 0.8 AU) (**Figure 3A**, left panel). At t24, the cells harboring P*abrB*’*gfp_Bte_*AAV do not present any fluorescence (Supplementary Figure S2). The shape of the histograms suggests that the P*abrB*’*gfp_Bte_*AAV fusion is expressed in a homogeneous fashion in the population (only 1 peak is visible). The right panel of **Figure 3A** recapitulates the data for both experiments as kinetics of median fluorescence intensity and shows that the peak of P*abrB*’*gfp_Bte_*AAV expression was reached between t-1 and t0. Microscopy pictures of P*abrB*’*gfp_Bte_*AAV-expressing cells harvested at t0 and at t2 support the flow cytometry data (**Figure 3B**). These results show that the P*abrB*’*gfp_Bte_*AAV transcriptional fusion is expressed during exponential phase and that we can visualize a decrease in its expression when the cells enter the transition phase.

### *abrB* Expression Is Activated in Cells That Have Entered the Necrotrophic Pathway

**FIGURE 4:**
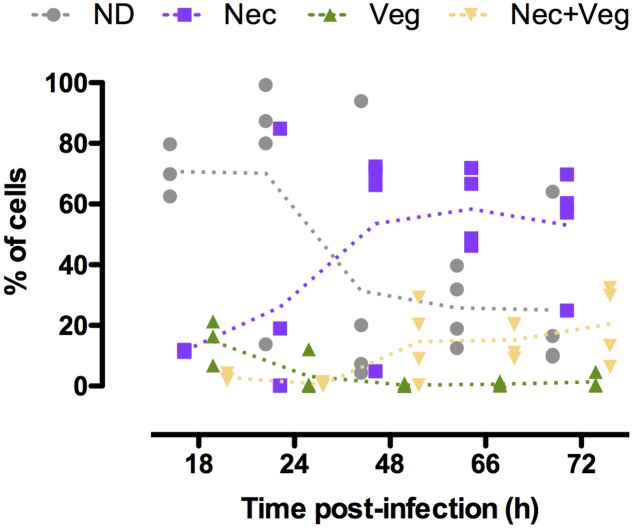
*abrB* and *nprA* promoter activities in cells isolated from insect cadavers. Flow cytometry analysis of *B. thuringiensis* cells harboring pP*abrB*’*gfp_Bte_*AAV-P*nprA*’*mcherry_LGC_*. Bacteria were isolated from cadavers of *G. mellonella* larvae infected by intrahemocoelic injection and incubated at 30°C. Samples were harvested 18, 24, 48, 66, and 72 h after injection. The percentage of cells of each population discriminated on cytograms (as described in the Supplementary Experimental Procedures) are presented as a function of time. Each population phenotype is associated to a color as indicated on the graph: ND, cells that do not express any of the markers used; Nec, cells expressing the necrotrophic marker P*nprA*’*mcherry_LGC_* only; Veg, cells expressing the vegetative growth marker P*abrB*’*gfp_Bte_*AAV only; Nec+Veg, cells expressing both the vegetative growth and necrotrophic markers. Each symbol represents bacteria extracted from one larva. The dotted lines connect the mean values at each time point. These data are the result of two independent experiments.

We investigated the physiological state of *B. thuringiensis* cells during infection in order to characterize in more detail the composition of the population during this cycle. To achieve this objective we examined the state of the cells that did not activate the promoters previously used, in particular the necrotrophic reporter (Verplaetse et al., 2015). We infected *G. mellonella* larvae with *B. thuringiensis* cells harboring the P*nprA*’*mcherry*_LGC_ transcriptional fusion as well as the P*abrB*’*gfp_Bte_*AAV transcriptional fusion on the same vector. *nprA* is under the control of NprR, the regulator of the necrotrophic state, and reflects its activity (Perchat et al., 2011). We monitored the fluorescence of the cells harvested from the insect cadavers at various times following infection. The results are presented in **Figure 4**. The kinetics profile of the Nec+ population (i.e., cells expressing *nprA*) is similar to what has been published before (Verplaetse et al., 2015) with a low percentage of the population expressing *nprA* 18 h post-infection (pi) (mean value of 11%) that increases between 24 and 48 h pi (mean values of 27 and 68%, respectively) to reach a maximum 72 h pi (mean value of 74%). We observed that the vegetative P*abrB*’*gfp_Bte_*AAV reporter is expressed at 18 h pi in 13% of the population. Interestingly, less cells expressed it at 24 h pi (mean value of 4%). The percentage of cells in which P*abrB* is expressed increased at 48 and 72 h pi (mean value of 15 and 22%, respectively). The expression of P*abrB*’*gfp_Bte_*AAV is almost exclusively restricted to cells that have activated the *nprA* gene promoter from 48 to 72 h pi. **Figure 4** also shows that the proportion of cells that did not express the necrotrophic or the vegetative reporters changed from 71% at 18 h pi to 25% 72 h pi. In order to verify that the expression patterns observed were not due to loss or copy number heterogeneity of the plasmid during the infectious process, we infected *G. mellonella* larvae with *B. thuringiensis* cells harboring the pP*aphA3*’*_sf_gfp* vector carrying a transcriptional fusion between the promoter of a constitutive gene in *B. thuringiensis*, and the reporter gene encoding _sf_GFP. The results show that the fusion is expressed in a homogeneous fashion in the population 24 and 72 h pi (only 1 peak is visible) (Supplementary Figure S3). Furthermore, a previous study has reported that 100 and 99.3% of the bacteria harvested from insect cadavers still carried a vector with the same backbone 48 and 96 h post-infection, respectively (Verplaetse et al., 2015). This indicates that the phenotypic heterogeneity observed for the reporters mentioned above is not due to plasmid instability or copy number heterogeneity in *B. thuringiensis* during infection.

### Cells That Did Not Enter the Necrotrophic Pathway Are Viable

**FIGURE 5:**
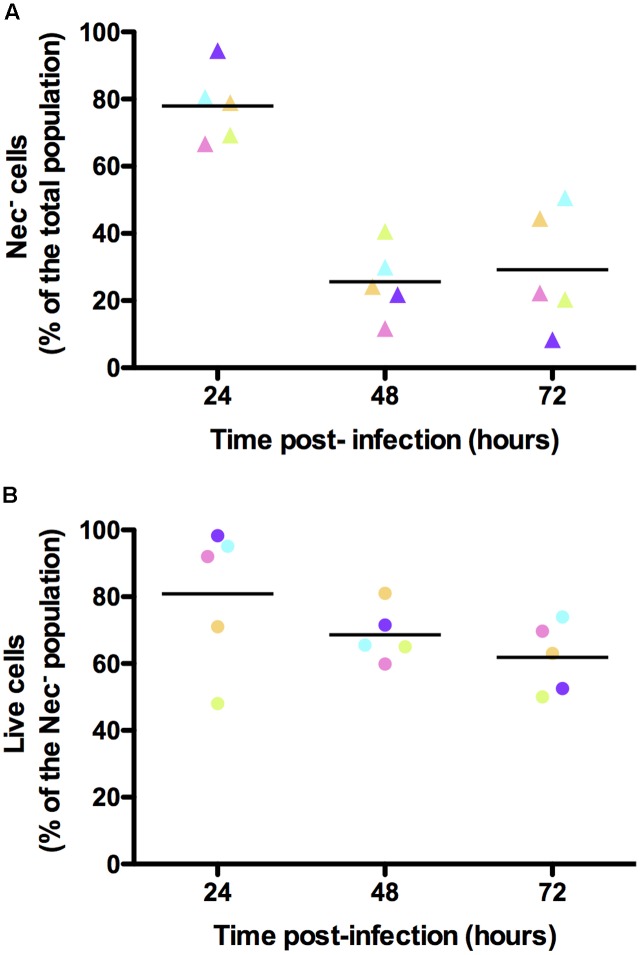
Viability assessment of the bacterial cells isolated from insect cadavers. Flow cytometry analysis of *B. thuringiensis* cells harboring pP*nprA*’*mcherry_LGC_* and stained with the SYTOX Green Dead Cell Stain. Bacteria were isolated from cadavers of *G. mellonella* larvae infected by intrahemocoelic injection and incubated at 30°C. Samples were harvested 24, 48, and 72 h after injection. The percentage of cells of each population discriminated on cytograms (as described in the Supplementary Experimental Procedures) are presented as a function of time. **(A)** Percentage of cells that did not enter the necrotrophic pathway (Nec^-^). **(B)** Percentage of cells that did not enter the necrotrophic pathway and that were considered live using the SYTOX Green Dead Cell Stain. Each symbol represents bacteria extracted from one larva. The color of the symbols for each time point in **(A,B)** indicates bacteria extracted from the same larva. These data are the result of two independent experiments and the lines represent the mean values at each time point.

In order to verify that the bacteria that did not express any of the reporters used were alive, we infected *G. mellonella* larvae with *B. thuringiensis* cells harboring the P*nprA*’*mcherry_LGC_* transcriptional fusion and incubated the samples harvested from the insect cadavers at various times following infection with the SYTOX Green Dead Cell Stain. We then immediately monitored the mCherry- and SYTOX Green-based fluorescence of the bacteria. The cells recovered at each time-point were mostly alive. Indeed 89, 79, and 84% of the bacteria were not stained by the SYTOX Green Dead Cell dye at 24, 48, and 72 h pi, respectively (Supplementary Figure S4). **Figure 5A** shows that in these conditions we recovered 78, 26, and 29% of Nec^-^ cells at 24, 48, and 72 h pi, respectively. In these samples, the SYTOX Green stain shows that 81, 69, and 62% of the Nec^-^ bacteria were live cells, respectively (**Figure 5B**). This indicates that the majority of the cells that did not express any of the reporters used were not dead.

## Discussion

Our initial goal was to characterize in more detail the composition of the *B. thuringiensis* population during the infectious process. We were interested in particular by the cells that did not express any of the reporters we used in a previous study, especially the necrotrophic state reporter (Verplaetse et al., 2015).

To begin to answer this question we constructed a genetic tool to enable us to follow gene expression fluctuations using a fluorescent reporter. We used the *ssrA*-mediated peptide tagging system (for a review Karzai et al., 2000) that was published for the first time by Andersen et al. (1998) as a tool to destabilize GFP in order to follow transient gene expression in bacteria. Destabilizing the GFP would reduce the fluorescence of the cells harboring this construct. Thus, it was also necessary to improve its fluorescence in *B. thuringiensis* cells. GFP improvement has been described for other bacterial species (Veening et al., 2004; Overkamp et al., 2013) and even bacteria of the *B. cereus* group (using *gfpmut1* as the original template) (Sastalla et al., 2009). We designed a new construct –*gfp*_Bte_– based on the _sf_*gfp* allele mainly because of its fast-folding properties (Pedelacq et al., 2006; Milde, 2008). The *B. thuringiensis* cells expressing *gfp_Bte_* were 20-fold brighter than the ones harboring the initial construct. The best improvement resulted from the codon optimization. Destabilization of the protein was then accomplished by adding a degradation tag to the GFP_Bte_. The resulting GFP_Bte_AAV rendered the cells almost as fluorescent as the ones without the tag, but with a half-life of approximately 15 min compared to at least 7.5 h for the original construct. This destabilization method has been adapted by various laboratories to different bacterial species (for example Blokpoel et al., 2003; Hentschel et al., 2013; Mouammine et al., 2017). However, to our knowledge, this system has been only used in very few and elegant studies to monitor fluctuations in gene activity in an infection model (Nielsen et al., 2010; Laughlin et al., 2014).

We fused the *gfp_Bte_*AAV reporter gene to the promoter of *abrB* and associated it to the P*nprA*’*mcherry_LGC_* transcriptional fusion to determine if the bacteria that did not go through the necrotrophic state during the course of infection were vegetative cells. We saw that the promoter of the *abrB* gene was active throughout the infection cycle, at least from 18 h pi to 72 h pi. At 18 h pi a large number of bacteria did not express either reporter. We cannot exclude that these cells expressed the P*abrB*’*gfp_Bte_*AAV fusion during the early stage of the infection, considering the unstable nature of the fluorescent protein and the fact that at this time point, the bacteria have reached a growth plateau after a period of active multiplication (Dubois et al., 2016). The proportion of vegetative cells and of undetermined cells then diminished as the proportion of necrotrophic cells increased. This was expected as the necrotrophic marker is a stationary phase marker. However, we did not expect the cells that went through the necrotrophic state to activate the P*abrB* promoter. The stability of the mCherry protein [half-life of more than 50 h (Verplaetse et al., 2015)] is a double-edged property. It allows us to follow the cells that went through the necrotrophic state but we do not know when -or if- they stopped expressing the necrotrophism genes. Using a fluorescent protein whose spectral properties change with time could help solve this issue (Terskikh et al., 2000). We hypothesize that the cells that activated P*abrB* were no longer in a necrotrophic state and that they resumed vegetative growth following a signal that they sensed. It was shown that the sporulating cells arose almost exclusively from necrotrophic cells in the host cadaver (Verplaetse et al., 2015). It is possible that re-activation of the vegetative state is a way of delaying sporulation in some cells. Sporulation is a costly and, at some point, irreversible process (Piggot and Hilbert, 2004). In *B. subtilis*, bacteria that have engaged in sporulation use toxins to kill non-sporulating cells (for a review Gonzalez-Pastor, 2011). This allows the non-committed sporulating cells to arrest sporulation and resume growth by using nutrients provided by the lysis of neighboring cells. Sporulation delay might be why the cells re-enter a vegetative state in this P*abrB*’*gfp_Bte_*AAV-expressing sub-population. The mechanism by which this could occur remains to be elucidated. Another hypothesis, which would not exclude the above mentioned one, would be that some of the spores germinated. Indeed, the spores, that originate from necrotrophic cells, retain an mCherry-based fluorescence (Verplaetse et al., 2015, 2016) and the P*abrB* promoter is activated while spores are in the process of germination (data not shown). It is likely that if nutrients become accessible, at least some of the spores will germinate.

**FIGURE 6:**
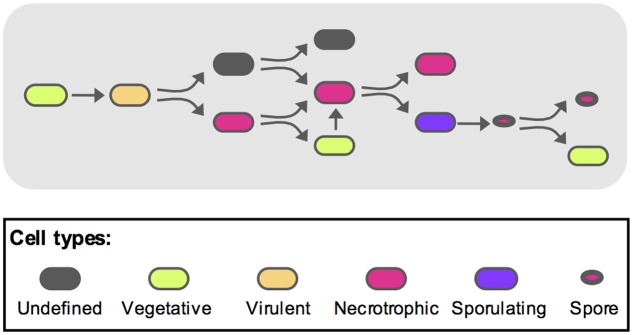
Model representing the different *B. thuringiensis* cell types during infection. Based on the model published by Verplaetse et al. (2015), we propose the following schematic to describe the various physiological states of *B. thuringiensis* during infection. The full description is given in the discussion section of the main text. Oblong shapes represent cells (i) in an undefined state (gray), (ii) in a vegetative state (green), (iii) displaying a virulent phenotype (orange), (iv) displaying a necrotrophic lifestyle (magenta) and (v) committed to sporulation (purple). Spores are represented as oval shapes with a red core.

To recapitulate these events and integrate them with the previously published data (Verplaetse et al., 2015), we propose the following model as schematized on **Figure 6**. During the early steps of infection, the bacteria activate the vegetative state marker while multiplying. After the virulence stage and death of the host, the bacterial number has reached a plateau and part of the population will turn on the necrotrophic genes. Among these bacteria, some will re-enter a vegetative state. These cells will subsequently have to activate the necrotrophic genes before sporulation can occur in part of this sub-population. Some spores are then able to germinate and resume vegetative growth. During this infectious cycle, some bacteria do not fit in any of the categories mentioned above. Indeed, the cells that did not go through the necrotrophic state did not appear to activate the P*abrB* promoter. About 20% of the population are still in an undetermined state. However, we have shown that about 70% of these bacteria are viable. This suggests that these cells could be dormant. Dormancy has been studied in Gram-positive and Gram-negative bacteria as a successful survival strategy (Rittershaus et al., 2013; Verstraeten et al., 2016). However, these species were non-sporulating. In sporulating bacteria, the spore is the ultimate form of dormancy. Nevertheless, a quiescent physiological state would constitute an alternative to the complex process of sporulation. Indeed, once the cells are committed to sporulation, they would not be able to return quickly to a vegetative state if competing bacteria invaded their niche. And bacteria in such a quiescent state could rapidly return to exponential growth, compared to germination, if favorable conditions were encountered, or might respond to a different resuscitation signal than that of germination. Many questions remain to be answered, such as the ability of these cells to grow back from this state. Their infectious capacity as well as their gene expression profiles during infection should also be investigated to determine if they can reproduce the parental phenotypes. We are now in the process of pursuing this research further.

## Author Contributions

Conceived and designed the study: LS and DL. Designed the experiments: LS. Performed the experiments: SBR and LS. Analyzed the data: LS, SBR, and DL. Wrote the paper: LS.

## Conflict of Interest Statement

The authors declare that the research was conducted in the absence of any commercial or financial relationships that could be construed as a potential conflict of interest.
