# Supplementary material for: Analysis of abrB Expression during the Infectious Cycle of Bacillus thuringiensis Reveals Population Heterogeneity
Source: Front Microbiol. 2017 Dec 12;8:2471. doi: 10.3389/fmicb.2017.02471 (PMC5732988; doi:10.3389/fmicb.2017.02471)
Supplement: Supplementary file 1 [file Data_Sheet_1.docx]

**Analysis of *abrB* expression during the infectious cycle of**

***Bacillus thuringiensis* reveals population heterogeneity**

**Samia Ben Rejeb, Didier Lereclus and Leyla Slamti***

Micalis Institute, INRA, AgroParisTech, Université Paris-Saclay,

78350 Jouy-en-Josas, France

***Corresponding author:**

Leyla Slamti

Micalis Institute, INRA,

78350 Jouy-en-Josas

France

00 33 1 34 65 23 82

[leyla.slamti@inra.fr](mailto:leyla.slamti@inra.fr)

**Experimental procedures**

**DNA manipulations**

Plasmid DNA was extracted from *E. coli* by a standard alkaline lysis procedure, using Promega kits. DNA fragments were purified using Promega kits. Chromosomal DNA was extracted from *B. thuringiensis* cells, harvested in mid-exponential growth phase, using the Qiagen Puregen Yeast/Bacteria kit. Restriction enzymes, T4 DNA ligase, Standard Taq DNA polymerase and Phusion high-fidelity DNA polymerase were purchased from New England Biolabs and used as recommended by the manufacturer. The oligonucleotide primers (Table S1) used for PCR amplification were synthesized by Sigma-Aldrich. PCR was performed with a 2720 Thermak cycler (Applied Biosystems). All constructs were systematically verified by PCR followed by sequencing of the region of interest. Nucleotide sequences were determined by Beckman Coulter Genomics.

**Flow cytometry**

For GFP- and STOX Green-based fluorescence, we used a solid blue-laser emitting at 488 nm combined to a 500-nm long pass dichroic mirror and a 527-nm band pass filter (512–542) (FL1 Channel). For mCherry-based fluorescence, we used a solid yellow-laser emitting at 561 nm combined to a 585-nm band-pass filter (FL4 channel). The analyses were performed using logarithmic gains and detector settings, adjusted on a sample of reporterless cells, to define cellular autofluorescence. Gating on FSC⁄SSC was used to discriminate bacteria from the background. For each sample, 20000 gated events were measured. Data were collected and analyzed with the FlowMax software (Sysmex Partec, France) and overlays were generated with the Weasel 3.3.3 software (WEHI, USA).

For each time point, to identify positive and negative populations on bi-parametric cytograms, we applied the 98 % division line for each fluorescent marker, *i*.*e*. we set the threshold on the reporterless strain so that 98% of the population gave a fluorescence intensity below the threshold. Cells giving a fluorescence signal above the threshold were considered positive. We cannot exclude that for a reporter expressed at a low level, a few positive cells might give a fluorescent intensity similar to that of the reporterless cells and be included in the negative population.

**Figures**

**Figure S1. Flow cytometry analysis of the *B. thuringiensis* cells harboring the empty vector pHT304.** The cells were grown in LB and harvested at various times during growth that can be identified with the time map on the graph. The X-axis of the histogram is the fluorescence intensity in arbitrary units (A.U.) in logarithmic scale. The Y-axis represents the cell count.

**Figure S2. Expression of the *abrB*-driven *gfp_Bte_*AAV gene *in vitro*.** Flow cytometry analysis of the *B. thuringiensis* cells harboring the empty vector pHT304 (grey areas) or pP*abrB*’*gfp_Bte_*AAV (lines). The cells were grown in LB and harvested at t-1 (light grey area and purple line) and at t24 (dark grey area and green line). The X-axis of the histogram is the fluorescence intensity in arbitrary units (A.U.) in logarithmic scale. The Y-axis represents the cell count.

**Figure S3. Expression of the P*aphA3*-driven _sf_*gfp* gene *in vivo*. (A)** Flow cytometry analysis of *B. thuringiensis* cells harboring the empty vector pHT304 (grey areas) or pP*aphA3*’_sf_*gfp* (lines). Bacteria were isolated from cadavers of *G. mellonella* larvae infected by intrahemocoelic injection and incubated at 30°C. Samples were harvested 24 (purple line and light grey area) and 72h (green line and dark grey area) after injection. The X-axis of the histogram is the fluorescence intensity in arbitrary units (A.U.) in logarithmic scale. The Y-axis represents the cell count. The experiment was realized once and the histograms are representative of 3 replicates. **(B)** Median fluorescence intensity of the same cells represented as a function of time. Yellow dots, *B. thuringiensis* cells harboring the empty vector pHT304; Green squares, *B. thuringiensis* cells harboring pP*aphA3*’_sf_*gfp*. The experiment was realized once. Each dot/square represents a replicate. The dotted lines connect the mean values at each time point.

**Figure S4. Viability assessment of the bacterial cells isolated from insect cadavers.** Flow cytometry analysis of *B. thuringiensis* cells stained with the SYTOX Green Dead Cell Stain. Bacteria were isolated from cadavers of *G. mellonella* larvae infected by intrahemocoelic injection and incubated at 30°C. Samples were harvested 24, 48, and 72h after injection. The percentage of cells that were considered live using the SYTOX Green Dead Cell Stain are presented as a function of time. Each symbol represents bacteria extracted from one larva. The grey and black dots represent 2 independent experiments and the lines represent the mean values at each time point.

**Table**

**Table S1.** Oligonucleotides used in this study

| **Name** | **Sequence** |
| --- | --- |
| sfgfp1 | gaagatcttaggaggatgattatttatg |
| sfgfp2 | ggggtacccaggaaacagctatgaccatg |
| sfgfpBt1 | tcggtctctcatgtcaaaaggagaggaattg |
| sfgfpBt2 | ggggtaccttaatgatgatgatgatgatgaga |
| sfgfpBtcomGBt1 | tcggtctctcATGAATGGGATTGAAATTTTTGCGatgtcaaaaggagaggaattg |
| gfpLAA | ggggtaccttaagcagctaaagaaagattgttttgtttgccatgatgatgatgatgagatc |
| gfpLVA | ggggtaccttaagctactaaagaaagattgttttgtttgccatgatgatgatgatgagatc |
| gfpAAV | ggggtaccttatactgctgcagaaagattgttttgtttgccatgatgatgatgatgagatc |
| gfpASV | ggggtaccttatacagatgcagaaagattgttttgtttgccatgatgatgatgatgagatc |
| Xyl10 | tcccccgggggcgcgccGAAATAAAATGCATCTGTATTTG |
| PU | cgccagggttttcccagtcacg |
| PabrB-F-XbaI | gctctagagctcgatatctttatcatc |
| PabrB-R-AscI | tggcgcgccctaaataaatagtttataagtttc |
| PabrB-F-NcoI | CATGCCATGGggggatgtgctgcaaggcgatt |
| gfpBteAAVin-BglII | GAAGATCTttatactgctgcagaaagattg |
| Term-R-BglII | GAAGATCTtgctgcataaaaaacgccc |
| NprA-F-NcoI | CATGCCATGGcgcggaaagggttttttcaa |
| PkanHind1 | cccaagcttgaaccatttgaggtgatagg |
| PkanBam2 | cgggatcccttttctacagtatttaaag |
